# Machine and deep learning meet genome-scale metabolic modeling

**DOI:** 10.1371/journal.pcbi.1007084

**Published:** 2019-07-11

**Authors:** Guido Zampieri, Supreeta Vijayakumar, Elisabeth Yaneske, Claudio Angione

**Affiliations:** 1 Department of Computer Science and Information Systems, Teesside University, Middlesbrough, United Kingdom; 2 Healthcare Innovation Centre, Teesside University, Middlesbrough, United Kingdom; Chalmers University of Technology, SWEDEN

## Abstract

Omic data analysis is steadily growing as a driver of basic and applied molecular biology research. Core to the interpretation of complex and heterogeneous biological phenotypes are computational approaches in the fields of statistics and machine learning. In parallel, constraint-based metabolic modeling has established itself as the main tool to investigate large-scale relationships between genotype, phenotype, and environment. The development and application of these methodological frameworks have occurred independently for the most part, whereas the potential of their integration for biological, biomedical, and biotechnological research is less known. Here, we describe how machine learning and constraint-based modeling can be combined, reviewing recent works at the intersection of both domains and discussing the mathematical and practical aspects involved. We overlap systematic classifications from both frameworks, making them accessible to nonexperts. Finally, we delineate potential future scenarios, propose new joint theoretical frameworks, and suggest concrete points of investigation for this joint subfield. A multiview approach merging experimental and knowledge-driven omic data through machine learning methods can incorporate key mechanistic information in an otherwise biologically-agnostic learning process.

## Introduction

Today, the search for biological mechanisms at molecular scale can leverage an unprecedented amount of information. With the recent development of high-throughput technologies, data collection has received an enormous impulse that has radically changed the perspective toward molecular biology. The main protagonist of this shift is omic data—namely, experimental profiles with large coverage over multiple biological domains. Several levels of knowledge have become associated with emerging omic technologies [1–3]. The most widespread to date include DNA sequencing (genomics), microarrays and RNA sequencing (transcriptomics), DNA methylation and histone modifications (epigenomics), and protein or metabolite mass spectrometry (proteomics and metabolomics). As technology moves forward, its associated costs decrease, and a growing wealth of data is being generated. Omic data therefore provide direct and convenient access to genetic variability and cellular activity. Undoubtedly, these datasets can be useful only if processed and deciphered through appropriate analytical tools.

A fundamental tool for the inspection, interpretation, and exploitation of omic data is machine (and deep) learning, which has arguably fueled several leaps forward in recent research and is expected to increasingly drive it in the near future [4, 5]. Machine learning can be described as a set of algorithms that improve prediction accuracy through experience, given a certain processable input from which they are able to learn and generalize. Beyond their predictive power, their diffusion in bioinformatics and computational biology is also due to the limited assumptions they require compared with other statistical or computational approaches. This makes them essential in a number of tasks, ranging from the understanding of RNA folding to estimating the impact of mutations on splicing and from the exploration of gene expression profiles to reconstructing phylogenetic trees [6–9].

In parallel, the increase in data and knowledge has also favored the development of mathematical models for biomolecular systems. Contrary to data-driven approaches, hypothesis-driven analysis of large-scale omic domains typically remains prohibitive given the difficulty in pinpointing the underlying biological mechanisms. There are, however, some exceptions. Among the various approaches, constraint-based modeling (CBM) of metabolism is receiving a huge impulse thanks to its wide scope and flexibility, enabling mechanistic insights into the genotype–phenotype environment relationship via integration with omic data [10]. With recent advances in technology, we are now able to reconstruct large-scale metabolic reaction networks of prokaryotic and eukaryotic cells, and genome-scale metabolic models (GSMMs) are constantly increasing in number and variety across all life kingdoms [11–15].

These two computational frameworks have mostly been used in isolation, having distinct research communities associated with them. However, we believe that their complementary characteristics and common mathematical bases make them particularly suitable to be combined. Several works implemented this idea in various ways and were partially surveyed before [16, 17]. Nevertheless, a comprehensive and systematic overview on this subject is lacking. In this work, we first review the existing approaches for integrating machine learning and CBM by compiling a thorough record of previous studies based on a combined classification of the two frameworks. Then, we suggest possible future research lines to develop new methodological approaches at the intersection of the two fields.

We therefore aim at providing a comprehensive and systematic catalog of existing interactions between CBM and machine learning while distinguishing between the various methodological and applicative aspects concerned. In general, the central idea is that GSMMs can be used to generate an additional omic layer: the so-called fluxomic data. The multiomic learning considered here then integrates this newly generated omic with the ones already available. For instance, concatenation of two datasets following normalization is a viable option. However, we will describe why this may not be the best approach in practice. Although it is outside of the scope of this work, we also remark that other computational techniques have successfully been used to build on CBM approaches and study the multiomic nature of various organisms. These include Bayesian [18] and metaheuristic optimization algorithms [19, 20], as well as methods drawn from the theory of games [21], graphs [22], Markov chains [23], and information [24].

In the following sections, we first concisely summarize the rationale and scope of machine learning and CBM of metabolism. Next, we review and classify previous studies in which these two frameworks were combined. Finally, we discuss similarities and differences among their mathematical bases, evaluate the advantages and limitations of computationally generating omic information, and outline aspects that have not been explored so far. To distinguish among the different types of mathematical models considered, throughout the text we will use the term “data driven” to refer to machine and deep learning models, whereas “knowledge driven” will refer to constraint-based models. If the meaning is intended to be more general, we will simply use the term “biological model.” Overall, we show that mining and integrating experimental and GSMM-generated multiomic data with machine learning techniques can unveil unknown mechanisms in a sample-specific manner, hence identifying relevant targets for biotechnology and biomedicine. Compared with approaches applying machine learning to omic data directly, we believe that a multiview approach merging experimentally and GSMM-generated omic data can include key mechanistic information in an otherwise biology-agnostic learning process.

## Data-driven exploration of biomolecular systems

The key problem in an increasingly omic-based biology is the difficulty in extracting knowledge from large and complex datasets. This task can be conveniently tackled through machine learning algorithms, many of which can be adapted to specific settings and omic types. A number of recent developments in the application of machine learning to problems in molecular biology and biomedicine have been critically analyzed in previous surveys, along with their limitations and challenges [4–9, 25–27]. Here, we concentrate on recalling the main characteristics of basic methods, with a focus on those suited for the simultaneous analysis of heterogeneous data.

### Types of machine learning approaches

A fundamental distinction in machine learning is between “supervised” and “unsupervised” learning approaches. In supervised learning, the goal is to predict one or more targets associated with a given sample. For instance, pathogenicity resulting from mutations can be predicted starting from the sequence as a continuous risk score or a discrete risk class. Broadly speaking, supervised learning methods can be subdivided into two main categories: classifiers, which aim to predict sample classes (e.g., pathogenic versus nonpathogenic variants), and regressors, whose task is to estimate numerical quantities (such as pathogenicity risk level). Several methods, such as support vector machines (SVMs) or artificial neural networks (ANNs), can be used to solve both classification and regression problems.

In contrast, unsupervised learning allows the exploration of data collections by deconstructing variation or correlations among samples. Unsupervised learning approaches are largely classified as either association algorithms, which uncover latent rules or trends in data, or clustering algorithms, which partition samples based on their inherent and often hidden characteristics. Owing to the large volume of omic data, its condensation or simplification can prove to be useful in order to facilitate its interpretation. The most popular approaches for data dimensionality reduction are (1) principal components analysis (PCA), which reduces data into low-dimensional representations summarizing maximum variance among variables; (2) factor analysis, which decomposes data based on latent relationships describing the correlation between variables; and (3) matrix factorization, which breaks down data matrices into denoised constituents. For instance, nonnegative matrix factorization (NMF) has been used to infer the ecological interaction networks of different gut microbial communities, starting from high-dimensional metagenomic samples [28]. Finally, as regards clustering approaches, the most widespread ones fall within the *k*-means and hierarchical clustering families, but many other algorithms are available with several applications [29].

### Machine learning for multiomic data

A single type of data usually offers a partial view on biological complexity and limits our understanding of it. Data-integration methods can facilitate the combined analysis of multiple omic datasets, which may be heterogeneous, in order to more closely represent genotype–phenotype relationships [1, 2, 30–34]. Data may be generated starting from the same samples through different omic measurements, or even with different omic measurements across different samples measured in the same system. As omic domains are inherently interconnected, signals missing from a single dataset can be compensated for in a multiomic data–driven model, therefore decreasing the likelihood of false negatives. At the same time, the mutual reinforcement of heterogeneous omic signals can limit false positives.

**Fig 1:**
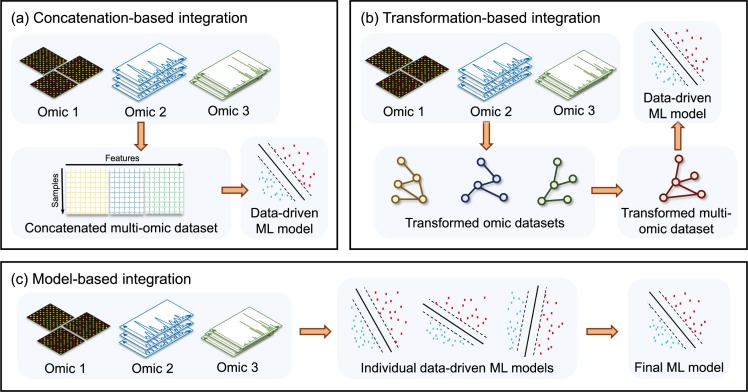
Omic data–integration methods in machine learning. Multiview omic data–integration methods can be classified into three main domains. (a) Concatenation-based (early-stage) integration involves combining all omic data into one large matrix before applying ML methods to obtain a data-driven model. (b) Transformation-based (intermediate-stage) integration involves applying data transformations to obtain a uniform format, which can then permit the combination into one fused dataset. (c) Model-based (late-stage) integration involves obtaining individual machine learning models separately for each dataset before combining the outcomes rather than combining data prior to the learning phase. ML, machine learning.

Most successful large-scale data-integration approaches are metadimensional methods, which simultaneously span multiple data sources and can cope with variable inputs [2]. They are broadly categorized into “concatenation-based,” “transformation-based,” and “model-based” integration, whose general characteristics are displayed in Fig 1. Alternatively, they are also called early-, intermediate-, and late-stage integration methods, respectively. In the machine learning context, algorithms dealing with data from multiple heterogeneous sources are referred to as “multiview” or “multimodal” learning algorithms [35, 36].

Concatenation-based integration (Fig 1A) fuses multiple data types together by concatenating data matrices into a single comprehensive matrix. Next, a learning algorithm is applied to this combined matrix. An advantage of this approach is the relative ease of applying statistical methods to any final data matrix. However, combining multiple matrices together can be challenging because of differences in scaling or inherent biases of each data type. Normalization techniques can be used to ensure that data of different orders of magnitude converge on the same scale, but differences in noise and variance can still affect the results [37], and as such, this kind of approach can lack reliability. Moreover, a data reduction step may be necessary if too many variables make the analysis infeasible.

Transformation-based integration (Fig 1B) converts each dataset into an intermediate form such as a “graph” or a “kernel matrix” (i.e., a matrix describing a precise, mathematically defined similarity among observations) [38]. The integration of the two datasets is then performed at the level of transformed data, hence resulting in an integrative graph or kernel matrix, which is used in the learning phase. This approach has the advantage of preserving the original properties of the data and the capability to combine virtually any data structure or format by applying the appropriate transformations. The main disadvantage is the difficulty of detecting interactions among different sources, missing cross-omic correlations and therefore resulting in hard interpretation.

Model-based integration (Fig 1C) generates machine learning models from each dataset and subsequently combines them to produce a final data-driven model. This kind of integration can have even larger flexibility compared with the transformation-based approach. For instance, in patient-centered studies, it is possible to combine models coming from various groups of patients for which different data sources have been analyzed. However, this strategy can miss interaction among different data types as well. Furthermore, it is particularly sensitive to overfitting, so it is recommended when the data pool is extremely heterogeneous.

All these strategies are commonly applied to heterogeneous datasets obtained from different experimental sources. However, there are also computational methods for generating data on the omic levels for which empirical means are inadequate. In particular, we concentrate on CBM of metabolism, as described in the following section.

## Constraint-based analysis of metabolic networks

Metabolism is one of the major biological components that coparticipates with the genotype in composing the phenotype. Metabolites can generate signals that are received at other omic levels, whereas metabolic feedbacks can compensate or modify genetic and environmental signals through complex nonintuitive routes [39, 40]. Unfortunately, omic-scale metabolite probing is still immature and suffers from major limitations. The main obstacles are high biochemical heterogeneity and concentration variations that can occur within subsecond timescales and span several orders of magnitude [41, 42]. In turn, metabolic reaction fluxes cannot be directly measured at large scale, and their estimation from indirect measurements presents even more challenges [43].

### Genome-scale metabolic models

Despite these experimental difficulties, metabolism remains the domain in molecular biology with the vastest knowledge, accumulated over the past century. Reconstructions of entire metabolic reaction networks have immediately followed after completing the first genomes in the late 1990s [44, 45]. GSMMs are mathematical representations of such networks and their relationships with associated enzymes and encoding genes, comprising the metabolic functionality of a cell [46]. A vast range of computational methods have been developed upon the framework of GSMMs to investigate interactions between genotype, environment, and phenotype [17, 47, 48]. Acting as integrative platforms for multiomic data, they can also help identify nonintuitive phenomena in metabolism [49]. Importantly, they also enable evaluation of the complete metabolic state of cell populations even when metabolome profiling is infeasible.

The mathematical framework of GSMMs is grounded on two physical assumptions. The first assumption is mass and charge conservation, which guarantees that the total mass of produced substrates equals the total mass of those consumed. Second, the system must be at steady state, meaning that internal metabolite concentrations do not change over time. The steady-state assumption differentiates CBM from the modeling based on ordinary differential equations. The latter allows the study of metabolic systems in dynamical conditions, but it is computationally expensive and requires detailed knowledge of initial metabolic conditions and kinetic reaction coefficients. For these reasons, it is only feasible for small systems and therefore cannot capture long-range phenomena or general metabolic reprogramming. Conversely, GSMMs are restricted to steady-state conditions, but they can span the entire cellular metabolism or even multicellular communities [50].

**Fig 2:**
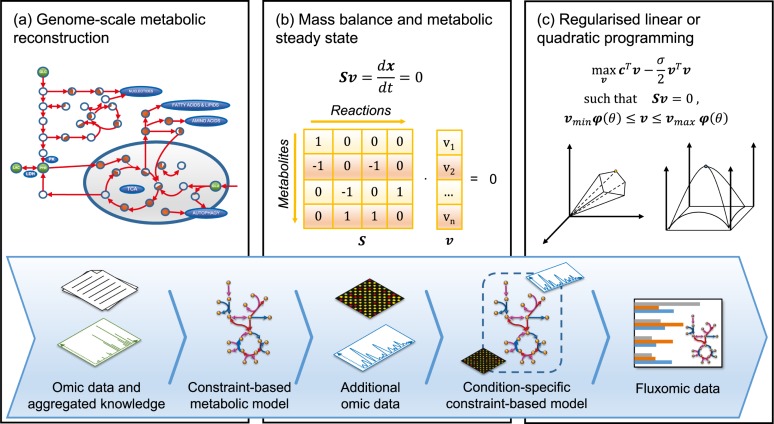
Constraint-based data integration and fluxome generation. (a) Constraint-based metabolic modeling begins with the construction of a manually curated GSMM recording all reactions taking place in the network. (b) Coded within the structure of a GSMM is the stoichiometric matrix **S**, denoting the involvement of metabolites in each reaction. Constraints are applied to the model to identify a given metabolic goal, represented as the objective function **c**, and linear or quadratic optimization is used to maximize or minimize this objective. The steady-state assumption (**Sv** = 0) sets the product of the stoichiometric matrix **S** and flux vector **v** as invariant. (c) To compute a unique flux distribution, the objective function can be regularized by subtracting a concave function from it. In addition to **v** being restricted between default lower and upper limits (v_*min*_ and v_*max*_), external multiomic data *θ* can be used to further constrain fluxes using the mapping function *φ*(*θ*), hence driving the output toward condition-dependent solutions. GSMM, genome-scale metabolic model.

Modeling fluxes can be crucial for gaining a better understanding of both metabolic activity and wider biological phenomena [10]. At a reaction and pathway level, flux balance analysis (FBA) is currently the most widely used tool to estimate the flow of metabolites in metabolic networks [46]. FBA allows determination of the flux configuration that yields maximal or minimal rate through one or more target reactions. In its basic form, it is mathematically defined as a linear optimization problem targeting a subset of reaction fluxes (Fig 2). Usually, when no other obvious cellular objective is involved, the maximization of biomass is considered as a reasonable goal not only for bacteria under evolutionary pressure but also for cancer cells under a proliferative regime [51]. For other types of cells, identifying the true objective is still a challenge; therefore, biomass is commonly taken as a reasonable proxy. Various FBA variants take into account further biological constraints or regularizations and are defined as quadratic, mixed-integer, or multilevel programs [52].

### Condition-specific constraint-based models

In a typical constraint-based metabolic model, fluxes are the variables whose values have to be determined. Because there are usually a greater number of reactions than metabolites in a GSMM, the problem is underdetermined—meaning that multiple solutions can satisfy it. In order to determine biologically meaningful solutions, it is often necessary to further refine the model by applying additional biological, physical, or chemical constraints. For example, these may account for enzyme capacity and promiscuity, spatial occupation, metabolite sequestration, and multiple levels of gene, transcript, and protein regulation [53]. Constraints derived from experimental data are particularly useful, as they are employed to build GSMMs that directly reflect observed biological conditions (e.g., those in particular tissues or pathological states).

The development of condition-specific or context-specific GSMMs constitutes a further data-integration framework, as shown in Fig 2. In this case, the process starts from raw data and knowledge on cellular physiology that are aggregated and converted into a GSMM. Although in the early phases of this field, global reconstructions were built by long manual efforts to aggregate and make sense of scattered information, methods to partially automate this process are now available, and in principle, they can be used to construct hundreds of knowledge-driven models [54–56]. General-purpose GSMMs can then be used as scaffolds onto which omic data are mapped during the successive integration, thus obtaining newly refined models with additional constraints. Mapped data can be transcriptomic, proteomic, and metabolomic profiles or information on splice isoforms or codon usage, as implemented in a number of works and software packages [57, 58]. Depending on the external data introduced, it is possible to generate GSMMs that reflect specific properties or states of particular tissues, cell types, microbial strains, or even individual cells.

Transcriptional profiles are the most popular omic to build context-specific GSMMs via an array of methods utilizing different contextualizing criteria [17, 59]. Switch-based methods utilize a gene expression threshold to turn off reactions associated with lowly expressed genes, thereby pruning the metabolic network. Conversely, valve-based methods map the transcriptional information on the constraint-based model in a continuous fashion. There are instead fewer approaches focused on the integration of proteomic and metabolomic data [60]. Commonly, algorithms such as iMAT [61], INIT [62], and METRADE [20] provide a framework for integrating both gene and protein expression data, with IOMA providing the opportunity to integrate proteomic and metabolomic data [63]. However, more specific approaches for protein data are being developed. For instance, a method known as GECKO constructs a GSMM with enzymatic constraints using kinetic and omic data [64]. This is achieved by expanding the stoichiometric matrix of the GSMM to include rows representing enzymes and columns representing enzyme usage in reactions, whereas enzyme kinetics (k_cat_ values) is modeled by pseudostoichiometric coefficients in this matrix. Constraining protein abundance in this way has the effect of significantly reducing flux variability and improving the accuracy of the predictions. Methodology and applications for condition-specific GSMMs have been reviewed in detail elsewhere [53, 59, 65, 66].

## Combining constraint-based analysis and machine learning

The integration of CBM of metabolism with machine learning is based on two key ideas. The first is that genetic and environmental perturbations propagate in a nonlinear fashion through metabolic networks and assume patterns on a reaction flux level that may be used to gain mechanistic insights into several research questions. The second is that GSMMs can act as both an analytical framework to represent biological systems and generators of information to be mined. In other words, flux solutions obtained by a GSMM can be treated like additional numerical data (another omic layer) and analyzed via learning algorithms. With the knowledge-driven metabolic model being set, the information extracted from it may depend on the task of interest and on the variables deemed relevant. As a result, it is possible to leverage the whole array of techniques defined on CBM [47] (see the "Constraint-based analysis of metabolic networks" section). Additionally, constraints at the metabolic level can be used to enhance the learning in multiomic settings, as explained in this section.

**Fig 3:**
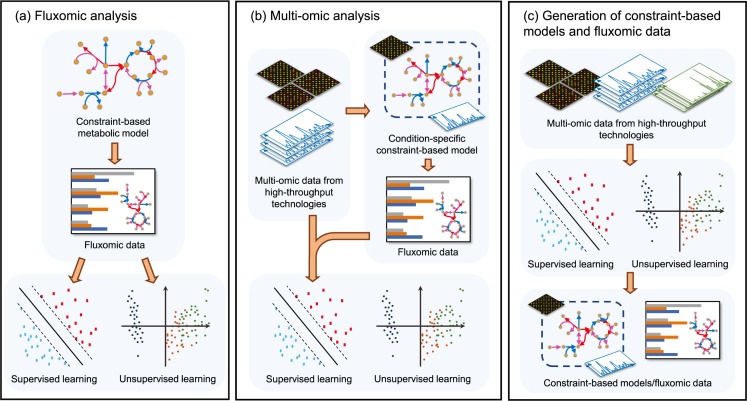
Multiomic data analysis by combination of constraint-based modeling with machine learning. (a) Fluxomic analysis involves FBA or related techniques performed on a general-purpose GSMM, from which the flux data obtained can be used as input for unsupervised or supervised machine learning. (b) To improve the accuracy of machine learning predictions, multiomic datasets are obtained using high-throughput analytics—e.g., transcriptomics (DNA microarrays, RNA sequencing), proteomics (2D gel electrophoresis, stable isotope labeling, mass spectrometry), or metabolomics (NMR spectroscopy, isotopic labeling, LC-MS, GC-MS). As these datasets are obtained from different sources, they must undergo several preprocessing stages such as filtration and normalization to maintain synchronicity, account for variance, and reduce noise. Condition-specific knowledge-based models are generated by introducing these multiple datasets into GSMMs to obtain more precise flux estimations, from which machine learning techniques can be applied to infer biologically relevant patterns in the data. (c) Alternatively, machine learning can be directly applied to single- or multiomic datasets to produce or improve GSMMs or fluxomic data. FBA, flux balance analysis; GC-MS, gas chromatography–mass spectroscopy; GSMM, genome-scale metabolic model; LC-MS, liquid chromatography–mass spectroscopy; NMR, nuclear magnetic resonance.

Despite these potential advantages, such integrated methodologies have remained confined to a few studies so far. In this section—to the best of our knowledge—we outline the existing examples of integration between machine learning and CBM grouped based on the task type as shown in Table 1 and Fig 3.

**Table 1:** Overview of previous studies that integrated CBM and machine learning, grouped by task type.

Study	Data integration approach	Machine learning component	CBM component	Task
**Supervised fluxomic analysis**				
[67]	-	Regularized multinomial logistic regression	FBA	Prediction of growth conditions
[68]	-	Bagging SVM, random forest	FVA, gene deletion	Inhibitory drug side effect prediction
[69]	-	ANN	FBA, gene deletion	Prediction of xylitol production
[73]	-	SVM, ANN, NMF	FBA	Prediction of bacterial ecological niches
[72]	-	Random forest	dFBA	Prediction of ecological interactions
[71]	-	Discriminant analysis	Elementary flux modes	Identification of distinguishing metabolic patterns between conditions
[70]	-	PCA, SVM, elastic net, random forest, XGBoost, kNN, ANN, ensemble learning	FBA	Estimation of titer, production rate, and yield of microbial factories
**Unsupervised fluxomic analysis**				
[74]	-	Hierarchical clustering	FBA	Characterization of epistasis in yeast metabolism
[76]	-	PCA	Random sampling	Decomposition of metabolic flexibility
[77]	-	PCA	Elementary flux modes	Identification of metabolic patterns
[75]	-	Hierarchical clustering	FBA	Exploration of ecological interactions
[78]	-	PCA	Stoichiometric constraints	Identification of responsive pathways
[71]	-	PCA	Elementary flux modes	Identification of metabolic patterns in dynamic conditions
**Supervised multiomic analysis**				
[79]	Concatenation based	SVM	FBA, reaction deletion	Reaction essentiality prediction
[83]	Constraint based	Kernel kNN	Maximization of consistency between reaction activity and gene expression	Drug target prediction
[80]	Concatenation based	Random forest, logistic regression	FBA	Genetic interactions prediction in yeast
[87]	Constraint based, concatenation based, model based	RNN, LASSO regression, ensemble learning	FBA	Cross-omic states prediction in *Escherichia coli*
[89]	Constraint based	Decision trees	TFBA	Estimation of kinetic parameter range and identification of key enzymes
[81]	Concatenation based	SVM-RFE	FCA	Prediction of gene essentiality
[88]	Transformation based	Sparse-group LASSO	Extreme currents	Identification of disease-deregulated pathways
[86]	Constraint based, concatenation based	Elastic net regression, PCA, GLM	Bilevel FBA	Prediction of lactate production in CHO cells
[90]	Model based	ANN, autoencoder	FBA, gene deletion	Phenotypic predictions in *E*. *coli* based on multiomic data
[84]	Constraint based	Elastic net regression	Bilevel FBA	Identification of polyomic predictors of aging
[85]	Constraint based	Elastic net regression	Geometric FBA	Identification of disrupted pathways in *Pseudomonas putida* mutants
**Unsupervised multiomic analysis**				
[91]	Constraint based	Bayesian factor modeling	Bilevel FBA	Prediction of temporal pathway activation in *E*. *coli*
[84]	Constraint based	Hierarchical clustering, *k*-means clustering	Bilevel FBA	Polyomic characterization of aging
[85]	Constraint based	PCA	Geometric FBA	Identification of biomarkers for rhamnolipids biosynthesis
[92]	Constraint based, model based	ANN	Stoichiometric constraints	Interpretation of gene expression data in *E*. *coli*
**Generation of constraint-based models and fluxomic data**				
[93]	-	kNN, decision trees, SVM	Stoichiometric constraints	Metabolic flux estimation based on general genetic and environmental conditions
[94]	Constraint based	PCA	FBA, Monte Carlo sampling	Characterization of engineered *E*. *coli* strains variation
[95]	Constraint based	PCA, linear regression	FBA	Metabolic flux estimation in dynamic conditions
[96]	Concatenation based, constraint based	Elastic net regression, random forest, neural networks, ensemble learning	FBA, pFBA, ME model	Prediction of proteomic data

The studies reviewed here and included in the table are grouped by task type: supervised or unsupervised fluxomic analysis, supervised or unsupervised multiomic analysis, generation of constraint-based models, and fluxomic data. Each study is annotated with the methodological building blocks related to the two computational frameworks (CBM and machine learning). Abbreviations: ANN, artificial neural network; CBM, constraint-based modeling; CHO, Chinese hamster ovary; dFBA, dynamic FBA; FBA, flux balance analysis; FCA, flux coupling analysis; FVA, flux variability analysis; GLM, generalized linear model; kNN, *k*-nearest neighbors; LASSO, least absolute shrinkage and selection operator; ME model, metabolism and gene expression genome-scale metabolic model; NMF, nonnegative matrix factorization; PCA, principal component analysis; pFBA, parsimonious FBA; RNN, recurrent neural network; SVM, support vector machine; SVM-RFE, SVM based on recursive feature elimination; TFBA, thermodynamics-based FBA; XGBoost, extreme gradient boosted trees.

### Supervised fluxomic analysis

The baseline case is when biological targets are predicted based solely on metabolic fluxes obtained from general-purpose GSMMs. The output of FBA or related techniques can then be fed to algorithms for supervised analysis without data integration being involved (see Table 1).

For instance, Sridhara and colleagues investigated whether bacterial growth conditions could be inferred from intracellular flux configurations [67]. Multinomial logistic regression was used in conjunction with least absolute shrinkage and selection operator (LASSO) regularization to relate growth conditions to simulated metabolic fluxes. The regression enabled prediction of growth conditions for a particular FBA solution by using internal metabolic fluxes as input, with regularization serving to select the most relevant fluxes and prevent overfitting.

In the context of human metabolism, integration of constraint-based models and machine learning has been shown to correctly identify side effects of inhibitory drugs with higher accuracy than baseline methods [68]. Drug-specific actions were simulated by in silico gene deletions, and the associated metabolic perturbations were estimated through flux variability analysis (FVA), whose results were fed to an ensemble SVM. Artificially reproduced metabolic alterations improved the results compared with a predictor used on drug biochemical structures. In a similar fashion, but for a different objective, a deep neural network and a differential search algorithm were applied to design gene deletion interventions in *E*. *coli* for the production of xylitol [69]. Also in this case, FBA coupled with artificial gene knockout served as a generator of genome-scale fluxomic data. Another recent study tested a flux-based data-driven approach for the prediction of titer, production rate, and yield across different bioprocessing settings [70]. Based on an ensemble of state-of-the-art machine learning techniques, flux features were shown to boost predictive accuracy in this scenario, typically characterized by sparse data.

Importantly, CBM and machine learning can be formulated as a joint problem by embedding stoichiometric constraints in a learning task. As an example of supervised method, a discriminant analysis technique based on metabolic network constraints—called dynamic elementary mode regression discriminant analysis (dynEMR-DA)—was defined to identify pathway activation patterns that best discriminate between experimental conditions [71]. The methodology expands the concept of elementary flux modes (EFMs)—which are the simplest paths in a GSMM that characterize the associated flux space—to dynamic conditions. The algorithm seeks to determine the EFMs that differ the most in terms of time evolution.

Expanding the analysis of fluxes to an ecological scale, DiMucci and colleagues developed an approach to predict interactions among bacterial species starting from temporal simulations of cocultures through dynamic FBA (dFBA) [72]. A random forest classifier was trained on binary vectors representing the exchange reactions in each GSMM, using dFBA relative yield predictions of cocultures with respect to independent cultures. This data-driven model allowed better generalization than the simple distance-based criterion commonly employed in microbial community studies and also allowed inferring the metabolic exchanges underlying the predicted interactions. In another ecological context, Chien and Larsen proposed that supervised classification of niches of bacterial species can benefit from the information generated by metabolic models [73]. They reconstructed GSMMs for 21 *Pseudomonas* species living in the endosphere and rhizosphere and simulated 12 media formulations in order to generate predictive features. A cross comparison of SVM, ANN, and NMF suggested that metabolic flux features may be more predictive than purely genomic features.

### Unsupervised fluxomic analysis

The exploration and statistical characterization of fluxomic profiles extrapolated from a GSMM can be of interest to shed light on the underlying physiology. In the absence of a well-defined biological target, unsupervised machine learning approaches can generally characterize correlation or variation across multiple samples. This allows clustering metabolic states or describing them in terms of sparser sets of variables.

This was first realized by Segre and colleagues, who exploited a GSMM to explore epistasis in yeast metabolism [74]. The task was accomplished by performing agglomerative clustering on the fitness landscape of single and double deleterious mutants for all genes involved in metabolism, for which the fitness was defined on FBA growth rate ratios. The analysis identified a widespread modular organization of genes into groups linked exclusively by buffering or aggravating epistatic interactions, leading the authors to extend the concepts of modularity and epistasis based on the observed intermodule connections rather than on intramodule properties. An analogous approach was employed in the context of gut microbiome ecology, in which Magnúsdóttir and colleagues performed a large-scale study on the ecological interactions among community members across a combination of Western or high-fiber diets and aerobic or anaerobic conditions [75]. Similarly, these interactions were evaluated in terms of hierarchical clustering of the relative growth between interacting and noninteracting pairs predicted through FBA. The microbes were then profiled based on their interactions, identifying three major subgroups enriched in species with different carbohydrate fermentation capabilities. Positive interactions were observed mainly among metabolically distant organisms, confirming independent studies.

Furthermore, dimensionality reduction techniques can be employed to deconstruct the entire flux space associated with constraint-based models, as done for *E*. *coli* [76]. In this case, PCA served to filter and synthesize the variation in biochemical reaction fluxes achievable by the metabolic network. Nontrivial cross correlations among pathway activities can be captured, and associated metabolic capabilities can be comprehensively evaluated in terms of imposed constraints.

Finally, as in the supervised scenario, the analysis of multiple flux profiles can benefit from constraining a learning objective with stoichiometric knowledge. Alternative hybrids of PCA and stoichiometric flux analysis, termed as principal elementary mode analysis (PEMA) and principal metabolic flux mode analysis (PMFA), extract flux modes generated by metabolic models that contribute the most significantly toward variance while penalizing deviations from the steady state [77, 78]. These methods are able to overcome some of the shortcomings of using general PCA for the statistical interrogation of flux distributions—e.g., the overlooking of reaction stoichiometry and the need for a predefined set of pathways. PEMA was also extended to analyze non-steady-state EFMs [71].

### Supervised multiomic analysis

When experimental data is available, it can be aggregated with CBM-generated fluxomes to build multiomic sets of features and predict targets of interest. Thanks to the peculiar advantages of each individual data-integration approach, there are multiple ways to combine them depending on the questions addressed and on the available resources. One-stage integration by machine learning methods is a possibility, as described in the Machine learning for multiomic data.

This strategy was first investigated to predict metabolic reaction essentiality in *E*. *coli*. FBA-like approaches coupled with artificial gene deletions can efficiently estimate essential reactions, although this often requires precise knowledge of nutrient availability in a given condition. The essentiality is usually evaluated merely based on the biomass accumulation rate, which may be an imprecise estimator in some cases. Plaimas and colleagues [79] investigated whether it could be possible to improve FBA predictions by combining the estimated growth rate with additional topological, genomic, and transcriptomic data. By using an SVM as classifier, they successfully verified an improvement in accuracy. An analogous approach was used by Szappanos and colleagues to predict positive and negative genetic interactions in *Saccharomyces cerevisiae* [80]. A random forest was trained with FBA-based fitness and genetic interaction scores in addition to a large array of gene-pair characteristics such as paralogy, protein annotations, protein interaction network topology, single deletant fitness, mRNA expression, quantitative phenotypic correlation, and compartment localization. Traditional features were shown to give low precision for the majority of gene interactions, whereas FBA-based features brought significant improvements in predictive precision and recall, indicating that genome-scale CBM captures relevant information that is missed by gene-level traditional features. The approach was tested again in the context of gene essentiality prediction by Nandi and colleagues [81], who instead employed flux coupling analysis (FCA) as feature generator to take gene adaptability into account in varying environmental conditions [82].

However, the metabolic capabilities of a cell population vary according to environmental and genetic conditions. For the sake of prediction, it is therefore important that metabolic information extracted by GSMMs reflects the differences between these conditions. This can be achieved through the creation of condition-specific metabolic models (see the "Condition-specific constraint-based models" section). This constraint-based integration was used for the first time by Li and colleagues to predict novel drug-reaction interactions in cancer [83]. They employed a linear programming model to enforce the agreement between gene expression and metabolic fluxes in order to determine fluxomic profiles relative to 59 cell lines, which were used for binary classification by a kernel *k*-nearest neighbor (kNN) model. A similar procedure was used to explore the molecular biology of aging [84]. Using the transcriptomic data from the CD4 T cells of 499 healthy participants, personalized CD4 T-cell metabolic models and their fluxomes were obtained with a continuous gene expression map [20]. Applying elastic net regression to these individual metabolic fluxes and the chronological ages of the individuals allowed establishing metabolic age predictors and their effect sizes. Using these polyomic predictors, the metabolic age of an individual could be defined and calculated, providing a basis for improved prediction of individual aging and life expectancy. A similar strategy was employed to metabolically and mechanistically evaluate the impact of synthetic mutations in *P*. *putida* starting from corresponding gene expression measurements [85].

Effectively learning from empirical omic profiles and associated GSMM-based metabolic states necessitates fully exploiting all the varieties of multiomic analysis methods. In this case, a two-stage integration can be achieved through the creation of condition-specific GSMMs and the subsequent machine learning–based data integration. This idea was used to predict the metabolic capabilities of Chinese hamster ovary (CHO) cells for diverse growth conditions [86]. In the study, it was shown that combining fluxomic and transcriptomic data in mammalian cells can provide a better estimation of secondary metabolite production, such as lactate. The pipeline includes building bioreactor-specific GSMMs and bilevel FBA optimization [20], which provided information on the metabolism associated with each growth condition. Later, both fluxomic and transcriptomic data were used to predict lactate accumulation with improved accuracy. Considering a wider omic array, Kim and colleagues developed a general framework for multiomic inference based on various machine learning methods [87]. Their platform can be used to perform cross-omic predictions among five biological layers: transcriptomic, proteomic, metabolomic, fluxomic, and phenomic. All of them are composed of experimental data aggregated from a number of studies, except the fluxomic layer, which is the result of condition-specific FBA following the integration of transcriptomic and proteomic data.

More sophisticated data-integration pipelines have also been developed. A study used a method similar to sparse-group LASSO to identify phenotypic extreme currents (ECs) based on a combination of metabolic network features and gene expression data [88]. Extreme pathways are subpathways (i.e., a subset of largely invariant pathways in the metabolic network that consistently yield steady-state flux), which are decomposed by linking them with a given phenotype. In other words, all ECs were associated with a gene set; based on gene expression data, those displaying a statistically significant association to a given clinical phenotype were identified. Uncertainty in the kinetic properties of enzymes is one of the main challenges in developing kinetic models of metabolism. Andreozzi and colleagues designed a strategy called in silico characterization and reduction of uncertainty in kinetic models (iSCHRUNK) to minimize such uncertainty [89], in which fluxomic and metabolomic data are integrated with a GSMM to create a thermodynamically consistent GSMM. Subsequently, decision trees are used to evaluate kinetic parameters. Finally, a recent work has used CBM to support an ANN. DeepMetabolism is an ANN method that integrates unsupervised pretraining with supervised training to build a deep learning model with the ability to predict phenotypic outcomes [90]. In its five-layer autoencoder, the first input gene layer was followed by two encoder layers (protein layer, phenotype layer), and the last two layers were decoders (reconstructed protein layer, reconstructed gene layer). Connections between the layers were regulated by biological priors, with FBA used to set the connectivity between the proteomic and the phenomic layer and therefore embed metabolic knowledge in the ANN architecture.

### Unsupervised multiomic analysis

Like in the supervised case, unsupervised algorithms can be applied on heterogeneous sets of experimental and GSMM-generated omic profiles. For instance, environmental condition–specific metabolic modeling was combined with statistical modeling by Angione and colleagues to estimate the metabolic pathway activation cascade triggered by different environmental stimuli [91]. The methodology was shown to better characterize the relationships among different pathways compared with static analysis, especially those occasionally interacting depending on the environmental conditions. In the same fashion, varying genetic conditions can be characterized in terms of associated changes on the metabolic level and potentially exploited in synthetic biology studies. For instance, decomposition of mutant-specific fluxomic profiles through PCA led to identifying novel biomarkers for rhamnolipids production [85]. Analogously, Yaneske and Angione utilized both agglomerative hierarchical clustering (AHC) and *k*-means clustering on transcriptomic data and fluxomic profiles in order to characterize the aging process in human [84]. Subsequent comparison of the clustering between transcriptomic and fluxomic data revealed that fluxomic profiles were better predictors of chronological age and age-associated metabolic biomarkers.

Moreover, metabolism and GSMMs can be used as a basis to understand underlying genomic variation. The Gene Expression Latent Space Encoder (GEESE) is a recently proposed approach [92] in which transcriptomic information is fed into a deep generative model (specifically, a variational autoencoder) combined with a GSMM. Initially, gene expression data is provided as an input to the autoencoder, returning reconstructed gene expression vectors that are then used to train an FBA approximator. The deep generative model is trained to minimize the loss between the fluxes obtained by passing the reconstructed gene expression through the approximated FBA and the fluxes generated by the real FBA while keeping the weights of this approximator constant. Based on this approach, latent patterns in gene regulation could therefore be identified while mechanistically accounting for downstream metabolic perturbations.

### Generation of constraint-based models and fluxomic data

Besides analyzing fluxomes generated via CBM, machine learning can be combined with CBM itself to acquire novel fluxomic information. For instance, a suite of different machine learning algorithms (SVM, kNN, and decision trees) was used to directly predict fluxomic configurations starting from genetic and environmental factors [93]. The training was performed by aggregating ^13^C metabolic flux analysis estimations with associated genetic and environmental information from a cohort of studies. In a second stage, the predicted flux outputs were adjusted to satisfy stoichiometric constraints using quadratic optimization in order to account for the flux balance and boost their accuracy.

Although in the previous paragraphs we have presented examples of machine learning applications on FBA outputs, data mining can even be used as a preliminary step to gain additional constraints for CBM. For example, Brunk and colleagues [94] applied a series of multivariate analysis methods (including PCA) on metabolomic data to better understand inner correlations and identify key metabolites influencing interstrain variation. Consequently, this enabled fixing sets of flux constraints inside the *E*. *coli* GSMM and achieving a better characterization of each culture phase. This strategy also allows estimating metabolic fluxes in conditions that are not directly accessible to FBA, such as in unsteady-state FBA (uFBA), in which multiple flux profiles associated with dynamic conditions can be predicted [95]. The underlying idea is to use PCA and linear regression to define constraints for an FBA model starting from metabolomics data. Because whole-metabolome measurements are generally difficult to achieve, uFBA also includes an algorithm to estimate unmeasured metabolite concentration differences on the basis of those that are measured. The obtained constraint-based model can be used for traditional FBA, FVA, or related analyses in dynamic conditions. Finally, an ensemble of methods were used to estimate enzyme catalytic turnover bounds for a whole *E*. *coli* GSMM, improving its predictions on proteome allocation compared with the integration of turnover rates measured in vitro [96]. It is interesting to note that, in this case, FBA solutions associated with random environmental conditions were also included in the supervised learning phase, corresponding to a fluxomic analysis as described in the "Supervised fluxomic analysis" section.

## Perspective

As detailed in the previous section, a number of data- and knowledge-driven workflows can be devised depending on the research goals and on the available resources. Ideally, multiomic settings appear the most promising for effectively grasping meaningful biological patterns, not only because of the well-known advantages of data integration but also considering the complementary characteristics of experimental and GSMM-based data. In the "Advantages and limitations of expanding the multiomic array in silico" section, we first articulate this point, highlighting the strengths and limitations of both omic types. In the "Emerging applications" section, we outline important scenarios to which we believe this multiomic machine learning framework could be applied and which are largely or entirely unexplored so far.

At the same time, many novel integrative methods could be developed given the variety of algorithms existing within the machine/deep learning and CBM frameworks. In particular, in the last section, we discuss two related aspects that we believe could inspire the design of novel integrative methods: the importance of interpretability in biological data-driven models and the connections of both CBM and machine learning to mathematical programming (see the "Building on common mathematical roots: Toward predictive and interpretable biological models" section).

### Advantages and limitations of expanding the multiomic array in silico

Because of their generation process, fluxomic profiles obtained through a GSMM provide an alternative and mechanistic perspective on the underlying biology compared with traditional omics. Both possess complementary benefits and drawbacks in scientific and operational terms, as outlined in this section, which make them particularly suitable for integration.

As previously pointed out, important differences exist first of all in terms of genetic coverage and prior knowledge [97]. Experimentally generated omic data can span vast portions of the genome, transcriptome, or proteome, despite the limitations of some technologies to achieve full coverage [98]. CBM is instead normally limited to metabolic networks, although extensions to other domains have been advanced [99, 100]. Second, generation of traditional omics requires no prior information, whereas GSMM construction assumes extensive knowledge of the metabolic system under consideration, although a semiautomated knowledge-driven model creation partially alleviates this burden [54–56]. On one hand, experimental data generation can be therefore more readily translated to new systems. On the other hand, experimental data is also prone to contain false-positive cues and can sometimes be superficial or ambiguous in its biological meaning. For instance, the high expression level of a gene does not necessarily lead to an increased enzyme activity if it is part of an enzymatic complex, as it would be limited by the expression of the other genes in the complex. Conversely, GSMMs are usually highly curated and provide a mechanistic description of biological processes, linking together genes, enzymes, metabolites, and reactions. GSMMs are therefore able to account for isozymes and enzymatic complexes through gene–protein reaction rules. Compared with annotations with an abstract structure, they can also describe the functional role of genes more precisely, as they provide a direct representation of biochemical processes. Despite their well-defined meaning, the scope and precision of fluxes generated in silico are, however, limited by the quality of the metabolic model used and by the available knowledge and understanding of a system, which may often be partial.

If experimentally generated omics are the first step toward a comprehensive understanding of living systems, the use of condition-specific GSMMs can therefore help contextualize and interpret them on a large scale. This fusion can also help identify gaps or inconsistencies in knowledge-driven models and maintain a comprehensive biological scope. Likewise, errors arising during experimental measurements might be mitigated through constraint-based integration, also controlling for biological soundness.

Furthermore, cost and time factors may motivate the integration of the two data types, albeit to varying degrees between constructing a GSMM or its condition-specific variants and the calculation of flux data. The initial building and curation of general-purpose knowledge-driven models can in fact be time-consuming and require up to months or even years, despite aid from computational pipelines [101]. However, if a baseline GSMM is already available, the creation of context-specific counterparts and associated flux solutions is generally fast through dedicated software. Besides, the generation of experimental omic data is notoriously cheaper than ever, but it remains a nonnegligible cost, especially when dealing with numerous samples. For cell systems with already-validated GSMMs, FBA and related techniques can therefore quickly provide an additional omic layer to integrate with the others at extremely low cost. This consideration is especially important in the case of large sample numbers, which are essential for machine learning methods to identify robust and biologically meaningful patterns.

There are nevertheless unsolved issues involving, to some extent, all omic data types and posing major limitations to studies based on them. In particular, we mention the quality of estimated biological phenomena and related biases. As previously mentioned, experimental measurement is subject to intrinsic noise and uncertainty that has to be corrected through appropriate normalization, and small numbers of technical replicates may undermine the statistical significance of the observed signals. Additionally, traditional omics are affected by sampling or technology-specific systematic errors [102]—in particular, batch effects [103]. In some cases, technology-specific issues can even compromise the overall data quality, like in the sequencing of PCR-challenging regions [98]. Besides, in silico calculation of fluxomes has to deal with uncertainty and bias on different levels as well. The steady-state assumption poses a limit to the kind of fluxomes that can be reasonably estimated [104], and in several situations, it may be unclear how to choose among multiple valid flux solutions. In addition to this, uncertainties arising in experimental settings may propagate to omic-based condition-specific GSMMs. As a result, external validation of FBA-predicted fluxes is generally required, at least on the level of cellular growth or most relevant pathways. Thorough GSMM evaluations are highly beneficial for the improvement of these platforms, but they have been conducted only in a limited number of systems, such as *E*. *coli* and *S*. *cerevisiae* [105–109]. However, as a consequence of the iterative refinement of GSMMs through the accumulation of new knowledge and data, their coverage and quality are rapidly increasing [14, 110].

All these points are very important to bear in mind for a correct and meaningful analysis and interpretation of the underlying biology. Overall, knowledge-driven fluxomic data relies on strong assumptions that require cautious evaluation to ensure biological soundness. At the same time, experimental data generation has to deal with issues that in some cases risk undermining any scientific conclusions. For these reasons, signals obtained from both experimental and GSMM-based omic studies should always be thoroughly evaluated through careful study design, appropriate statistical methods, and independent data (when available), without omitting negative results in downstream reports [111, 112].

### Emerging applications

Despite the challenges highlighted in the previous section, omic data analysis will probably remain fundamental in numerous contexts and spread to new ones. Given the complementary advantages of GSMM-generated fluxomes and other omic data, their integration therefore has the potential for many novel applicative scenarios. As long as steady-state metabolism is deemed relevant to the task at hand, CBM can be employed to extend the omic or multiomic data array, and machine learning techniques can be used to identify hidden patterns. For instance, metabolic engineering could enormously benefit from integrative biological models, which are more efficient and cost-effective than empirical trials [113] in terms of both pathway design [20, 85] and bioprocess parameters [70, 86, 114]. Further, CBM extensions for modeling dynamic conditions can overcome the intrinsic limitations of FBA and open the door to another range of applications. In spite of this, only a few studies have investigated this scenario so far, as visible in Table 1.

Our survey also shows that many previous studies dealt with bacterial systems—in particular, *E*. *coli*, which is arguably the organism with the most highly curated GSMMs. However, for several eukaryotic organisms—including human—constraint-based models are now available with increasing scope and precision, which constitute a promising platform for integrative biological models. Initial studies have demonstrated this in the context of human aging and disorders [84, 88], as well as for drug development [68, 83]. The accumulating wealth of data extracted from human tissues is a particularly valuable resource, which has however yet to be fully exploited through data- and knowledge-driven approaches. This union has the potential to unveil novel clinical biomarkers and drug targets if properly implemented in omic studies.

Moreover, there are emerging research areas that are likely to require strong analytical and automation skills in the near future. In particular, we refer to those applications that require GSMMs of growing size, such as for cell populations and microbial communities. At present, CBM can be used to describe multiple cell types, tissues [115], or even the heterogeneity within cell populations [116]. Given that no cell lives in isolation, all these models will be increasingly important to understand its interactions and behavior in larger systems. At the same time, focusing on individual cells is increasingly revealing essential in fields like cancer biology, in which single-cell technologies are being improved and expanded to new omic layers. Single-cell RNA-seq alone will make the amount of data generated scale up to the millions of samples, or even higher numbers [34]. The spread of these technologies can further fuel the emergence of a larger omic data era, with the associated challenges in terms of data analysis and interpretation. Even in this context, data- and knowledge-driven computational tools appear essential to cope with these challenges.

Finally, as mentioned previously, CBM is extensible to biomolecular domains other than metabolism. Efforts have particularly focused on integrated constraint-based models of gene expression and metabolism [99, 100]. The formulation and validation of novel constraints could aid in developing further methods for multiomic data mining, but at the same time, it poses challenges associated with the increase in knowledge-driven model size and heterogeneity.

In all these contexts, we believe that effectively combining machine learning and CBM allows achieving a richer and more meaningful mechanistic comprehension of inherently multiomic system. New integrative approaches are also expected to ultimately contribute to the progress of applicative fields such as biotechnology, bioengineering, and biomedicine.

### Building on common mathematical roots: Toward predictive and interpretable biological models

One last aspect of pressing importance concerns the trustworthiness of integrative data- and knowledge-driven models and their capacity to produce novel insights. Interpretability is a desirable property for any mathematical model, and it constitutes a particularly delicate and widespread criticality in machine learning. Indeed, most automatically generated models are complex and provide no direct explanation for their predictions. At the same time, interpreting results, generating hypotheses, and testing them is imperative to maintain scientific rigor [117]. As discussed before, in this context, model interpretation relies on model transparency and post hoc analyses [118]. Transparency refers to the human understanding of a whole model, a learning algorithm or their parts, variables, and parameters. For example, in multiple kernel learning, the weights assigned to input kernels can in some sense be regarded as their contribution to the given task. Alternatively, it is possible to infer relationships between input and output through additional algorithms or reasoning applied a posteriori. For instance, some methods can determine data samples whose predictions are similar, or they can compute local dependencies on input features.

Note that interpretability does not uniquely depend on the data-driven model form but also on the input preprocessing. A neural network trained on intuitively meaningful features learns a data representation that can be visualized and reasoned upon more easily than a linear model trained on heavily processed features.

It can be therefore argued that CBM constitutes a vehicle for obtaining biological knowledge in the form of coherent information equipped with mechanistic relations at a single-reaction level. From this point of view, the generation of flux data from a condition-specific GSMM can be regarded as an elaborate but transparent feature engineering step, in which a fluxome is the result of combining available omics with expert knowledge and mathematical optimization. Therefore, we believe that CBM could be the key to building more interpretable machine learning models—for instance, by providing variables of clear meaning [68, 72].

Perhaps even more importantly, paths for building more interpretable and mechanistically meaningful biological models exist also on a methodological level. In particular, it may be useful to consider that both machine learning methods and FBA-based approaches are grounded in mathematical programming (also called mathematical optimization), even though from distinct points of view.

In machine learning, optimization tasks target any cost function that is assumed to minimize the true predictive error and allow the final model to generalize. Like in traditional mathematical optimization research, this discipline seeks to formulate investigative questions in terms of tractable and scalable problems. In addition, other qualities such as easy implementation and interpretation are important, disregarding high accuracy and robustness across wide classes of problems [119]. Many of these goals are shared also by metabolic CBM. First, tractability and scalability remain of primary interest, especially with the growing size of GSMMs and microbial community models. Second, highly precise solutions are not indispensable, as long as they define the phenotypic state(s) associated with prior assumptions. In fact, the use of regularization is starting to be recognized as a standard in FBA, with the goal of identifying more realistic solutions, especially when the full flux distribution is used for inference or postprocessing [52]. Moreover, the ease in implementation and interpretation of FBA-related approaches is usually guaranteed by embedding physical, biochemical, or evolutionary assumptions.

This underlying connection can potentially be exploited to develop novel hybrid methods and provide a second way toward more transparent biological models. For instance, the learning problem can be formulated by integrating constraints borrowed from a knowledge-driven model. This idea has been already implemented both in the context of unsupervised fluxomic analysis [77, 78] and supervised fluxomic analysis [71], as described in the "Combining constraint-based analysis and machine learning" section. However, the existing case studies are limited, and more work is needed to understand how to effectively integrate mechanistic biological information in data-driven algorithms, especially given their variety and heterogeneity. Although challenging, the idea appears particularly promising if extended to multiview learning methods that would thereby fully leverage an expanded combination of data and knowledge.

## Conclusion

The use of machine and deep learning in computational and systems biology will keep growing in parallel with the rapid advancement of high-throughput omic technologies. However, extensions of current methodologies are needed to adapt to the heterogeneous, multidimensional nature of omic data. Here, we have explored the joint application of machine learning and genome-scale metabolic modeling in the context of multiomic analysis, evaluating strengths and pitfalls in developing hybrid methods that draw from both fields. Machine learning is a valuable tool for deconstructing biological complexity for the purposes of condensing high-volume multiomic datasets and extracting relevant outputs from them. In turn, CBM makes it possible to analyze metabolic activities associated with distinct properties or states specific to each cell, tissue, or community. This is achieved through multiomic data integration and the estimation of an additional (flux)omic layer that is closer to cellular phenotype.

CBM can provide ways to inject mechanistic knowledge within novel multiview methods, aiding in the achievement of data- and knowledge-driven analysis of biological systems. Given the increasing recognition of the importance of metabolism and mechanism-aware omic data analysis in a range of biomedical and biotechnological problems, we envisage that this multiomic machine learning approach could be useful to researchers across computational biology.
